# Supplementary material for: Creeping yeast: a simple, cheap and robust protocol for the identification of mating type in Saccharomyces cerevisiae
Source: FEMS Yeast Res. 2022 Mar 17;22(1):foac017. doi: 10.1093/femsyr/foac017 (PMC9202641; doi:10.1093/femsyr/foac017)
Supplement: foac017_Supplemental_Files [file foac017_supplemental_files.zip › Supplementary_Figure_1&legend-Arras_et_al.docx]

**Supplementary Figure 1: Time-lapse photography of the mating assay.** An **a**+α assay was set up as described in **Figure 1A** and photographs were taken every minute for 20 hours. Views at 0, 4, 8, 12, 16 and 20 hours show the formation and persistence of the creeping phenotype compared to a tight pellet for non-mating cells. For a video of the full time-lapse, see **Supplementary Movie.**
