# Supplementary material for: Creeping yeast: a simple, cheap and robust protocol for the identification of mating type in Saccharomyces cerevisiae
Source: FEMS Yeast Res. 2022 Mar 17;22(1):foac017. doi: 10.1093/femsyr/foac017 (PMC9202641; doi:10.1093/femsyr/foac017)
Supplement: foac017_Supplemental_Files [file foac017_supplemental_files.zip › Supplementary_Figure_2&legend-Arras_et_al.docx]

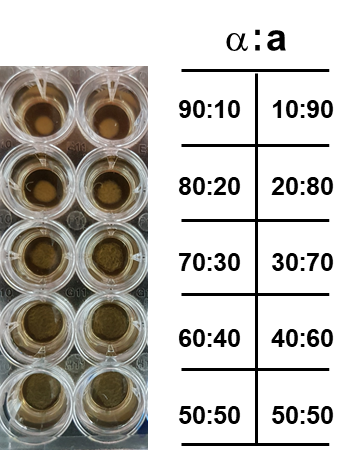


**Supplementary Figure 2: Symmetric formation of creeping phenotype with different ratios of each mating type.** Opposite mating type cells were mixed at the different ratios indicated in a final volume of 200 μL in a 96 well plate. The plate was photographed after 18 hours at 22˚C. The right column shows increased ratios in favour of mating type **a** cells, the left column shows increased ratios in favour of mating type α cells. This assay supplements **Figure 2C.**
