# Supplementary material for: Creeping yeast: a simple, cheap and robust protocol for the identification of mating type in Saccharomyces cerevisiae
Source: FEMS Yeast Res. 2022 Mar 17;22(1):foac017. doi: 10.1093/femsyr/foac017 (PMC9202641; doi:10.1093/femsyr/foac017)
Supplement: foac017_Supplemental_Files [file foac017_supplemental_files.zip › Supplementary_Figure_3_Legend.pdf]

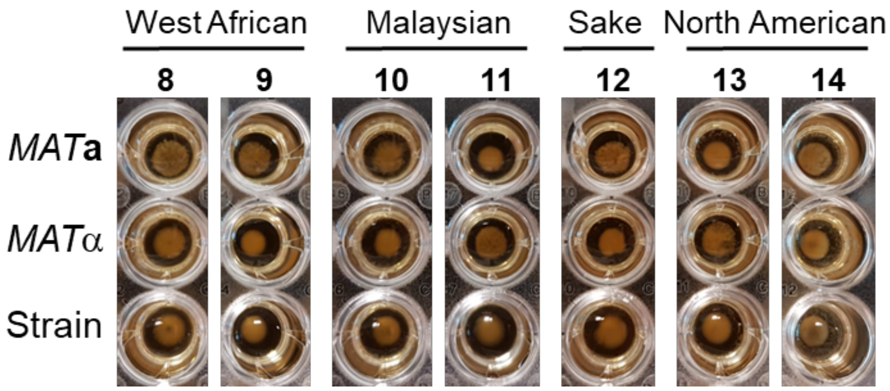

Supplementary Figure 3: Mating assay as in **Figure 3A** for an additional seven wild isolates that cover the West African, Malaysian, Sake, and North American non-mosaic *S. cerevisiae* clades described by Liti *et al.* (2009) as indicated. These strains were mixed with *MAT***a** and *MAT*α tester strains using standard assay conditions (YPD medium, OD_{600nm} 0.2, room temperature incubation) and were photographed after 5 days. The bottom wells (Strain) are negative controls containing strains 8 to 14 alone without any tester strain. The creeping phenotype is visible for strains 8, 9, 10, 12 and 14 crossed with *MAT***a** tester but not with *MAT*α tester. The opposite phenotype is visible for strains 11 and 13. All strains are described in **Supplementary Table 1.**
