# Supplementary material for: Creeping yeast: a simple, cheap and robust protocol for the identification of mating type in Saccharomyces cerevisiae
Source: FEMS Yeast Res. 2022 Mar 17;22(1):foac017. doi: 10.1093/femsyr/foac017 (PMC9202641; doi:10.1093/femsyr/foac017)
Supplement: foac017_Supplemental_Files [file foac017_supplemental_files.zip › Supplementary_Figure_4_Legend.pdf]

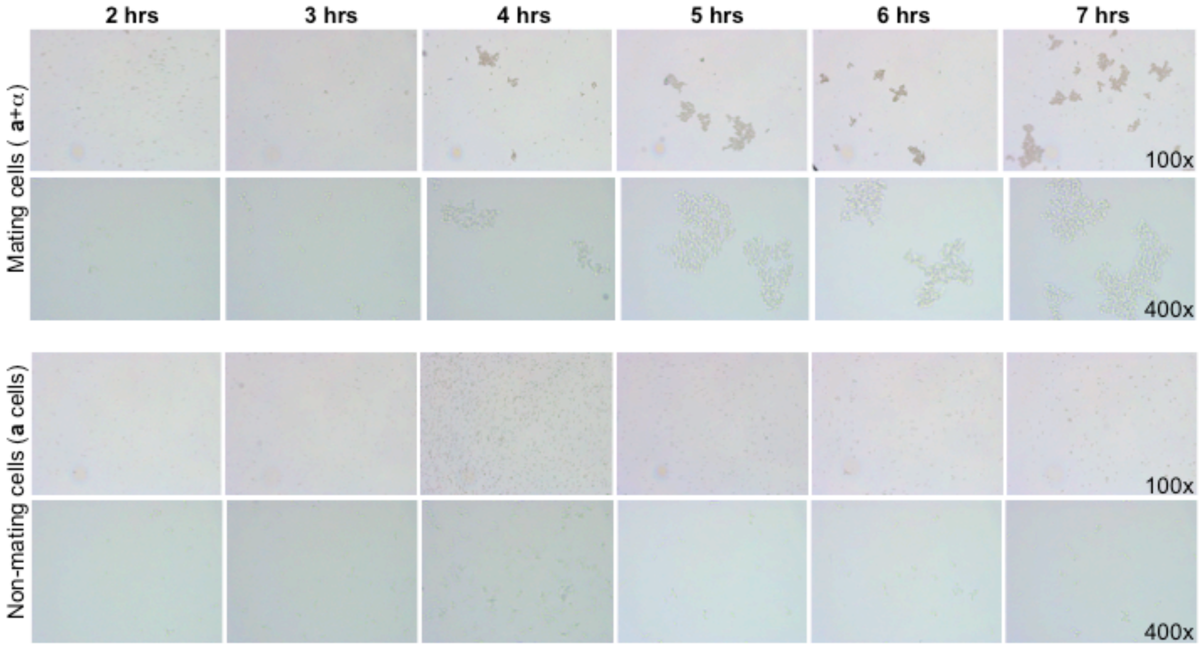

Supplementary Figure 4: Creeping cells aggregate over time. Full time course of mating and non-mating samples of **a**+α cells or haploid **a** cells observed under the microscope as described in **Figure 4**. Images were taken from 2 to 7 hrs after cells were mixed. Representative images at 100x and 400x magnification are shown.
