# Supplementary material for: Creeping yeast: a simple, cheap and robust protocol for the identification of mating type in Saccharomyces cerevisiae
Source: FEMS Yeast Res. 2022 Mar 17;22(1):foac017. doi: 10.1093/femsyr/foac017 (PMC9202641; doi:10.1093/femsyr/foac017)
Supplement: foac017_Supplemental_Files [file foac017_supplemental_files.zip › Supplementary_Figure_5_Legend.pdf]

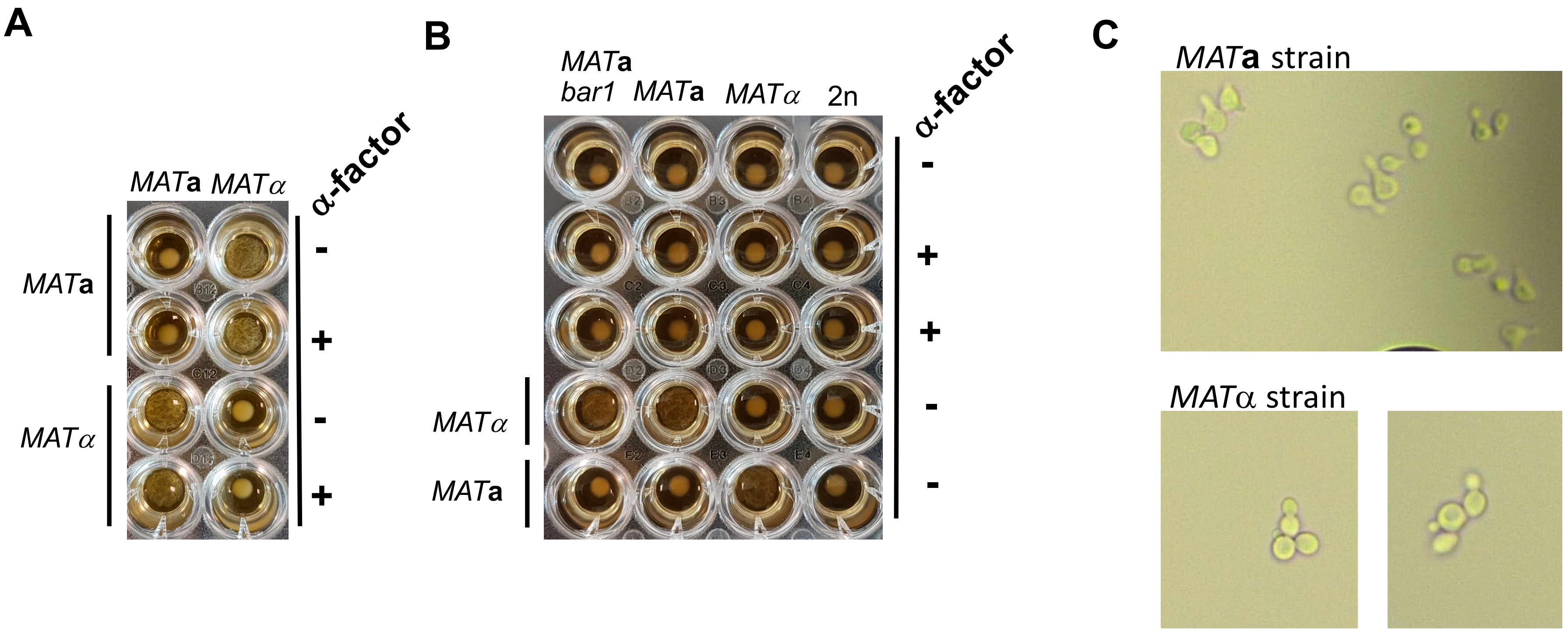

Supplementary Figure 5. No effect of α-factor addition on the creeping phenotype. (**A**) Addition of α-factor (50 μM; ZymoResearch) did not modify the creeping phenotype of mating cells and *MAT***a** cells treated with α-factor did not display a creeping phenotype when using standard assay conditions (YPD medium, OD_{600nm}=0.2, room temperature incubation). (**B**) A wider panel of strains were assayed individually (top three rows) and with mating partners as in (**A**) (bottom two rows), with (+) and without (-) α-factor. Here also *MAT***a** cells treated with α-factor did not display a creeping phenotype. A *bar1* mutant strain (*MAT***a** *bar1*) was included as α-factor is degraded by the *barrier* (Bar1) peptidase secreted by *MAT***a** cells (but not *MAT*α cells). This mutant also showed no creeping phenotype in the presence of α-factor. (**C**) The activity of α-factor was tested with a shmooing assay. 30 μL of a dense YPD overnight culture of each strain was inoculated into 5 mL of YPD, the cultures were grown at 30˚C with agitation until OD_{600nm}=0.2, then 190 μL of each culture was transferred to a 50 mL tube, treated with 10 μL of 1 mM α-factor (giving a final concentration of 50 μM) and incubated for 3 hours at 30˚C with agitation. The cells were observed for shmoo formation by microscopy. *MAT***a** (but not *MAT*α) cells displayed a shmoo phenotype, confirming the activity of the α-factor.
