# Supplementary material for: Creeping yeast: a simple, cheap and robust protocol for the identification of mating type in Saccharomyces cerevisiae
Source: FEMS Yeast Res. 2022 Mar 17;22(1):foac017. doi: 10.1093/femsyr/foac017 (PMC9202641; doi:10.1093/femsyr/foac017)
Supplement: foac017_Supplemental_Files [file foac017_supplemental_files.zip › Supplementary_Table_1.pdf]

Supplementary Table 1: Strains used in this study

Strain	Name	Genotype	Clade[a]	Reference
haploid *MAT***a**	NOY408-1b	*MAT***a** *ade2-1 ura3-1 his3-11 trp1-1 leu2-3, 112 can1-100*	Mosaic	Nogi et al. 1991
haploid *MAT*α	NOY408-1a	*MAT*α *ade2-1 ura3-1 his3-11 trp1-1 leu2-3, 112 can1-100*	Mosaic	
diploid (2n)	NOY398	*MAT***a***/ MAT*α *ade2-1/ ade2-1 ura3-1/ ura3-1 his3-11/ his3-11 trp1-1/ trp1-1 leu2-3, 112/ leu2-3, 112 can1-100/can1-100*	Mosaic	
1[b]	3582/ UWOPS87-2421	*MAT***a**, Δ*ho::HygMX*, Δ*ura3::KanMX*	Mosaic	Cubillos et al. 2009
2	3589/ UWOPS83-787.3	*MAT***a**, Δ*ho::HygMX*, Δ*ura3::KanMX*	Mosaic	
3	3593/ YJM981	*MAT***a**, Δ*ho::HygMX*, Δ*ura3::KanMX*	Wine/European	
4	3597/ DBVPG6765	*MAT***a**, Δ*ho::HygMX*, Δ*ura3::KanMX*	Wine/European	
5	3616/ BC187	*MAT*α, Δ*ho::HygMX*, Δ*ura3::KanMX*	Wine/European	
6	3620/ DBVPG1373	*MAT*α, Δ*ho::HygMX*, Δ*ura3::KanMX*	Wine/European	
7	3624/ L-1528	*MAT*α, Δ*ho::HygMX*, Δ*ura3::KanMX*	Wine/European	
8	3625/ DBVPG6044	*MAT*α, Δ*ho::HygMX*, Δ*ura3::KanMX*	West African	
9	3626/ NCYC110	*MAT*α, Δ*ho::HygMX*, Δ*ura3::KanMX*	West African	
10	3627/ UWOPS03-461.4	*MAT*α, Δ*ho::HygMX*, Δ*ura3::KanMX*	Malaysian	
11	3603/ UWOPS05-217.3	*MAT***a**, Δ*ho::HygMX*, Δ*ura3::KanMX*	Malaysian	
12	3630/ Y12	*MAT*α, Δ*ho::HygMX*, Δ*ura3::KanMX*	Sake	
13	3607/ YPS128	*MAT***a**, Δ*ho::HygMX*, Δ*ura3::KanMX*	North American	
14	3632/ YPS128	*MAT*α, Δ*ho::HygMX*, Δ*ura3::KanMX*	North American	
haploid *MAT*α	BY4742	*MAT*α *his3Δ1 leu2Δ0 met15Δ0 ura3Δ0*	Derivative of S288c lineage	Open Biosystems
haploid *MAT***a**	BY4741 YKO parental strain[c]	*MAT***a** *his3Δ1 leu2Δ0 met15Δ0 ura3Δ0*	Derivative of S288c lineage	Open Biosystems

[a] According to Liti et al, 2009.
[b] Strain 3582/ UWOPS87-2421 is reported as being mating type **a**, but we have shown that our version of this strain is mating type α, possibly as a result of a mis-labelling error.
[c] Yeast Knockout (YKO) library used in this study.

References

Cubillos, F.A., Louis, E.J., Liti, G. Generation of a large set of genetically tractable haploid and diploid *Saccharomyces* strains. *FEMS Yeast Res*. 2009. **9**:1217-1225.

Liti G., Carter, D.M., Moses, A.M., et al. Population genomics of domestic and wild yeasts. *Nature* 2009. **458**:337-341

Nogi, Y., Yano, R., Nomura, M. Synthesis of large rRNAs by RNA polymerase II in mutants of *Saccharomyces cerevisiae* defective in RNA polymerase I. *PNAS* 1991. **88**:3962-3966.
